# Dietary Determinants of Dental Caries Prevalence and Experience in Saudi Schoolchildren: Frequency versus Quantity

**DOI:** 10.1155/2022/5447723

**Published:** 2022-01-04

**Authors:** Abdulkarim Al-Zahrani, Mohammed Al-Qahtani, Mohammed Al-Barti, Eman A. Bakhurji

**Affiliations:** ^1^College of Dentistry, Imam Abdulrahman Bin Faisal University, Dammam, Saudi Arabia; ^2^Central Care Clinics, Alkhobar, Saudi Arabia; ^3^ADEED, Riyadh, Saudi Arabia; ^4^Department of Preventive Dental Sciences, College of Dentistry, Imam Abdulrahman Bin Faisal University, Dammam, Saudi Arabia

## Abstract

**Background:**

Sugar-added diet has been associated with increased risk of developing dental caries.

**Objective:**

To investigate the dietary determinants of caries prevalence and experience based on the frequency versus the quantity of consumption among Saudi schoolchildren.

**Methods:**

This is a cross-sectional study that invited 12- to 15-year-old intermediate school students (*N* = 3000) in four cities in the Eastern Province of Saudi Arabia. Data collection comprised a pretested questionnaire and clinical examination. Caries was diagnosed based on the World Health Organization (WHO) criteria and was recorded as a decayed, missing, filled tooth (DMFT). The questionnaire included questions regarding the consumption of different quantities and frequencies of multiple sugar-containing food items. Multiple logistic and linear regression models were used to assess the influence of sugar-containing food on the dental caries experience (DMFT) and prevalence of decayed teeth. SPSS version 20.0 was used to analyze the data at 5% significance level.

**Results:**

Of 3000 invited students, 2262 participated with a participation rate of 75.4%. Our study found that the frequency of soft drink consumption was significantly associated with increased caries prevalence (adjusted OR = 1.33, 95% CI 1.07–1.65) and experience (adjusted *B* = 0.46, 95% CI 0.16–0.76). The frequency of consumption of fruit juice was statistically significantly associated with increased caries experience (adjusted *B* = 0.48, 95% CI 0.16–0.79). The interaction terms between the frequency and the quantity of consumption of energy drinks, soft drinks, and sweetened milk were statistically significantly related to caries experience and prevalence.

**Conclusion:**

Although the frequency of consumption of sugar-containing drinks was more associated with caries experience and prevalence, the interaction between frequency and quantity was highly related to the prevalence and experience of dental caries.

## 1. Introduction

Dental caries remains the most common disease of the oral cavity that affects all age groups of different socioeconomic levels [1, 2]. Bacteria, time, susceptible tooth surface, and fermentable carbohydrates are the four main factors associated with the development of tooth decay [3, 4]. Lifestyle, socioeconomic status, smoking, xerostomia, reduced exposure to fluoride, and poor oral hygiene are also contributing factors to its widespread distribution [5, 6].

Monosaccharide and disaccharide dietary sugars are both present naturally or are added to food. Studies showed that natural sugars found in vegetables, grains, fruits, and also milk have no association with the development of dental caries [7–9]. Added sugars, however, show a strong association with the development of dental caries, and therefore, their intake should be controlled [10]. In 2002, it was recommended that free sugars should not exceed 10% of the total energy intake of diet [7]. In 2015, the World Health Organization (WHO) published guidelines regarding sugar intake, which recommended a reduction of sugar intake for adults and children alike [11]. Despite the efforts to reduce sugar intake among children and adolescents, the consumption of sugar-sweetened beverages has increased over the past three decades by more than 300%, and dietary sugar intake constitutes the largest source of added sugar in adolescents' diets [12].

The relationship between dental caries and sugar consumption is known to follow a dose-response curve [13]. However, few studies have investigated the association between the frequency and the amount of sugar intake in relation to dental caries. Some studies found that the quantity of consumption is more important [14–16]. Another study found that both frequency and quantity are of importance [17]. The relative significance of quantity compared with the frequency of sugar intake is difficult to evaluate because both factors are highly correlated, and an increase in one factor will result in an increase in the other [14, 18]. Yet, both the quantity and the frequency of consumption of sugar are found to be risk factors for the development of dental caries [7]. Therefore, the aim of this study was to investigate the influence of sugar-containing diet on caries prevalence and experience based on the frequency versus quantity of its consumption among Saudi adolescents.

## 2. Methods

This cross-sectional study was conducted in intermediate schools in Dammam, Khobar, Dhahran, and Qatif, in Eastern Province, Saudi Arabia, in January-February 2019. The target group was 12- to 15-year-old male and female students. A stratified, multistage sampling strategy was used for the selection of schools and students. Schools are distributed in the region by the Ministry of Education based on population density. The Ministry of Education randomly selected schools from the four major cities in the region (Dammam, Khobar, Dhahran, and Al-Qatif). The number of schools from each city was based on the size of the city, with more schools chosen from larger cities. A total of 16 schools were selected for this study (six schools in Dammam, four schools in Khobar, four schools in Al-Qatif, and two schools in Dhahran). Using simple random sampling, classrooms were selected from each school. Eligible students were selected from each classroom by random selection from a list. The students were included in the study if (1) they were attending public or private schools in Khobar, Dammam, Dhahran, or Qatif, (2) their parents or legal guardian consented in writing for their participation, (3) the student himself/herself agreed to be clinically examined, and (4) they had no health problems or had been on medication for the last six months. The students who did not return the written informed consent of their parents or legal guardians were excluded from the study. The study was approved by the Institutional Review Board at the Deanship of Scientific Research at Imam Abdulrahman Bin Faisal University, Dammam, Saudi Arabia (IRB-2015-02-189).

Prior to the start of the study, the sample size was calculated using the following assumptions: confidence interval = 95%, margin of error = 2%, percentage of adolescents with unfavorable dietary habits who have caries = 20%, total population = 20,000, and estimated response = 80% (https://www.checkmarket.com/sample-size-calculator). The required sample size for the study would be 2144, and the suggested number to invite would be 2680. All students in sixteen public and private schools were randomly approached to participate, and all students in these sixteen intermediate schools were asked to join the study (*N* = 3000).

Data collection included a questionnaire and a clinical examination. The clinical examination was based on the WHO criteria, using disposable examination mirrors under daylight in the schools. The DMFT index (decayed, missing due to caries, and filled teeth) was recorded for all permanent teeth. Five examiners had three sessions to train on the diagnostic criteria followed by a calibration session. The examiners had an acceptable level of agreement with an experienced gold standard examiner (weighted kappa ≥0.6). A kappa coefficient of 0.6 and above was considered adequate agreement (https://www.datanovia.com/en/blog/kappa-coefficient-interpretation/). A questionnaire was distributed to students. The questionnaire consisted of (1) demographical background variables (gender, age, parental education), (2) oral health practices (brushing, use of fluoride toothpaste, dental visits), and (3) information about dietary habits using the Food Frequency Questionnaire which is a validated questionnaire for use in adolescents [19, 20]. The FFQ had questions regarding the consumption of different dietary items such as candies, chewing gum, fruits, biscuits, fruit juice, sports drinks, energy drinks, soft drinks, unsweetened tea/coffee, and sweetened milk. Participants were asked about the frequency and the quantity of consumption of these dietary items. Study exposures were the frequency of consumption: ≤once a week versus ≥daily, and the quantity of consumption: ≤299 ml/day versus >299 ml/day. Samples of cans and bottles with different quantities were used to aid the students in identifying their quantity of consumption. The outcomes of the study were dental caries experience which was measured using the WHO criteria DMFT index and prevalence of decayed teeth.

Data were imported to SPSS version 20.0 (Armonk, NY: IBM Corp.) and analyzed at a 0.05 significance level. The main dependent variables were DMFT (caries experience) and decayed teeth (presence/absence) (caries prevalence). Descriptive statistics were calculated in percentage and frequency for categorical variables and mean and standard deviation for continuous variables. Statistical analyses involved univariate and multivariate logistic and linear regressions to assess the association between the frequency and the quantity of dietary consumption and dental caries prevalence and experience. Variables were included in the multivariate model if they are significant in univariate association. Interaction terms between the frequency and the quantity of the same dietary items were tested as well. Only those with significant interactions were added to the multivariate models. The collinearity of dietary items was tested to ensure that all variables entered in the linear multivariate model were not correlated. Also, a tolerance and variance inflation factor (VIF) test was performed to confirm the collinearity of variables. All dietary items in the present study had less than 2 in VIF, 0.2 of collinearity. Therefore, all dietary variables were entered in the multivariate linear models. All multivariate models were adjusted for oral health practices and demographics (gender, age, parental education, dental visits, and brushing habits) with a significance level of 5%.

## 3. Results

Of the 3000 students invited to participate in this study, 2262 returned the questionnaire and were examined (participation rate 75.4%).  Table 1 presents the demographic distribution, parental education, and caries prevalence and experience of the respondents. Fifty-seven percent of respondents were female, whereas the mean ± SD age of the adolescents in the study was 14.08 ± 1.3 years old. Most of the participated adolescents had less educated parents (58% of fathers and 64% of mothers had no or school education). The prevalence of caries in the study population was 75%, while the mean ± SD DMFT of the total sample were 4 ± 3.3 carious teeth.

**Figure 1:**
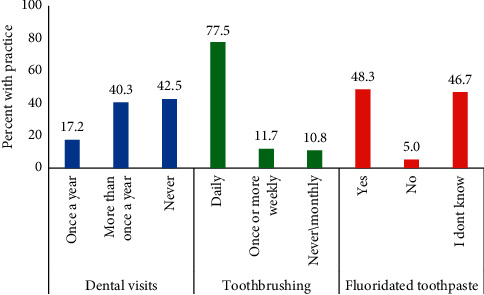
Distribution of oral health practices among study participants (*N* = 2262).

Figure 1 presents the distribution of participants' oral health practices. Forty percent of participants visited the dentist more than once in the past year versus 43% who had never visited the dentist before. Majority of the participants (77%) reported brushing their teeth daily, while only 48% reported using fluoridated toothpaste. Forty-seven percent do not know what kind of toothpaste they were using.

**Figure 2:**
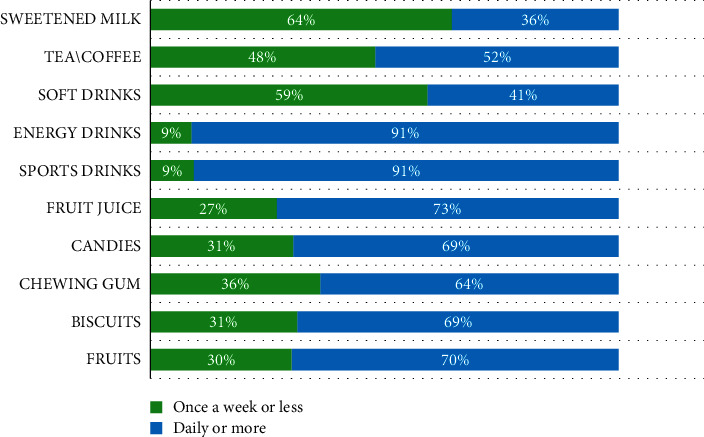
Distribution of the frequency of dietary item intake by study participants (*N* = 2262).

Figure 2 shows the distribution of frequency of intake of different sugar-containing items among the study participants. Energy and sports drinks were the most frequently consumed dietary items by 91% of participants. Fruit juice was daily consumed by 73% followed by candies (69%) and then chewing gum (64%). On the contrary, 70% of participants reported consuming fruits daily.

The univariate and multivariate logistic regression models predicting caries prevalence in adolescents are shown in Table 2. The overall multiple logistic model was statistically significant, with seven variables in both models. Regarding the frequency of diet consumption, chewing gum and candies were statistically significantly associated with caries prevalence (unadjusted OR = 1.25, 95% CI 1.03–1.52; OR = 1.25, 95% CI 1.02–1.53, respectively) in the univariate model. However, this relationship becomes nonstatistically significant in the multivariate model although the strength of association does not change much (OR = 1.15, 95% CI 0.94–1.42; OR = 1.08, 95% CI 0.87–1.33, respectively). More frequent consumption of sweetened beverages such as fruit juice (OR = 1.26, 95% CI 1.02–1.55), energy drinks (OR = 1.88, 95% CI 1.22–2.89), and sweetened milk (OR = 1.47, 95% CI 1.21–1.79) was statistically significantly associated with caries prevalence in univariate models. The quantity of consumption did not show evidence of an effect in univariate models except for energy drinks where higher quantity of consumption had almost two times the odds of developing carious lesions and that was statistically significant (95% CI 1.24–2.93). Interestingly, increasing the quantity of fruit juice and sweetened milk was not statistically significantly associated with developing carious lesions. This relationship is not evident in the multivariate model. After adjusting for demographics and oral health practices, although the more frequent consumption of fruit juice increased caries prevalence by almost 20%, it was not statistically significant. Only more frequent consumption of soft drinks was statistically significantly associated with higher odds of caries prevalence. The interaction of frequency and quantity of intake of energy drinks, soft drinks, and sweetened milk was statistically significant in the final multivariate model.

A similar pattern is seen in the model predicting caries experience (expressed in mean DMFT), as shown in Table 3. Using standardized B estimates, more frequent and higher quantities of consumption of energy drinks were the most predictive variables (standardized B estimates = 0.66 and 0.58), respectively, followed by more frequent consumption of soft drinks, fruit juice, and sweetened milk (standardized B estimates = 0.5, 0.48, and 0.42, respectively). However, more frequent consumption of fruit was the least predictive of having higher DMFT (standardized B estimates = −32). Oddly, more frequent consumption of sports drinks and consumption of higher quantity of fruit juice and sports drinks were not statistically significantly associated with DMFT in univariate models. After adjusting for demographics and oral health practices, only the more frequent consumption of fruit juice and soft drinks was statistically significantly associated with higher DMFT. A higher quantity of consumption of the same variables was not statistically significantly related with higher DMFT in the multivariate model. Similar to the prevalence of the caries model, the interaction terms of energy drinks, soft drinks, and sweetened milk showed statistical significance and were included in the multivariate model.

## 4. Discussion

This study investigated the influence of frequency versus quantity of several sugar-containing dietary items that constitute most of the Saudi adolescents' daily diet. The overall results of this study showed that the frequency of sugar intake was more associated with caries prevalence and experience than the quantity.

A significant association was found between sticky food, such as sugar-containing chewing gum, and the prevalence and experience of dental caries. On the contrary, nonsticky sugary items such as biscuits had a nonsignificant association with dental caries prevalence and experience. These findings were consistent with those of Alhabdan et al. who reported that chewing gum was significantly associated with dental caries among Saudi children attending primary schools and children aged 12–13 years [21, 22]. This might be related to the potential cariogenicity of sugar-containing gum and candy depending on their consistency, time of oral retention, and frequency of consumption [23]. However, the quantity and the frequency of the sticky sugary diet became irrelevant in multivariate models where sugar-containing drinks were more associated with the development of dental caries.

The association between the frequency of consuming sugar-sweetened beverages (SSBs) such as fruit juice, energy drinks, soft drinks, and sweetened milk and dental caries prevalence and experience was significant in univariate models. However, these relationships became nonevident in the multivariate models. A review of the literature which investigated the relationship between sugar and dental caries, from years 1856 to 2007, showed that it is not the quantity of sugar consumed, but it is the frequency which has a moderate significant relation with dental caries [24]. Only the consumption of fruit juice and soft drinks was strongly and significantly related to the development of dental caries. Similarly, the quantity of consumption of sweetened milk showed no association with dental caries prevalence or experience. According to a recent report of the WHO, in many countries, sugar-sweetened beverages, including fruit and milk-based sweetened drinks, are the primary source of free sugars in the diet [23]. In addition to their cariogenic potential, all carbonated drinks cause enamel erosion and thus increase the levels of dental caries [25].

The frequency of intake of sports and energy drinks was associated with dental caries prevalence, but not experience. On the contrary, there was no association between consumption of sports drinks and dental caries prevalence and experience. However, the interaction models for soft and energy drinks showed a significant relationship with dental prevalence and experience. Although the intake of sports and energy drinks has increased in popularity in recent years, a number of studies stated that sports drink was not directly associated with dental caries [26, 27]. However, there is no precise evidence supporting a relationship with dental caries, and therefore, the potential cariogenicity of these drinks should be carefully considered [28] and studied longitudinally for this age group.

Our study showed high frequency of intake of most sugar-containing food items especially energy and sports drinks, followed by fruit juice, candies, chewing gum, and biscuits. These dietary items contain high amount of added sugar. This high quantity of sugar in diet is addictive and is associated with binge eating and lack of self-control among adolescents [29]. Nevertheless, adolescents are more prone to emotional eating which usually leads them to more consumption of addictive food and beverages [30]. On the contrary, eating disorders that involve binge eating such as bulimia had been associated with dental caries in some studies among adolescents [31]. However, more recent studies did not find any associations between binge eating among adolescents and dental caries [32, 33]. Despite the conflicting evidence regarding binge eating as a result of eating disorders and dental caries, there is a dose-response relationship between sugar consumption and dental caries among adolescents [34].

Global and local healthcare systems have put tremendous efforts to improve oral health among young children and adolescents. Studies showed that supportive environments, along with productive policies, are associated with healthier choices of food [35]. In Saudi Arabia, the school nutrition policy banned many food items that can contribute to bad dietary habits or unhealthy outcomes for schoolchildren, such as soda, energy drinks, sports drinks, cakes, biscuits, chocolates, and chips [36]. Compliance of schools in different regions is variable, and more efforts to monitor and control school nutrition compliance are needed, especially among older students [37]. These dietary habits that can be achieved from school nutrition policies are expected to reduce the burden of dental caries among students [38]. Nevertheless, to have a greater impact, any oral health program implemented to improve the students' oral health must include parents and families, as the family has a great influence on the children's dietary behaviors [39].

A major strength of this study is the large sample size and the stratified multistage sampling strategy that included adolescents of four major cities in the region, and therefore, the results could be generalized to populations with similar demographical and social background. On the contrary, the present study may face few limitations. This is a cross-sectional study that investigated the influence of sugary diet on the development of dental caries without considering the longitudinal shift in behavior and time needed for dental caries to manifest. Therefore, a direct cause and effect relationship cannot be established. However, we have measured caries experience which reflects history of dental caries in the form of missing and restored teeth. This might give an insight into a temporal relationship between diet and dental caries. This study also relied on adolescents' self-reports of dietary intake and oral health practices which might have introduced recall bias and reporting of favorable behavior. However, due to the high consumption and significant association found in this study between consumption of sugary diet and dental caries, self-reporting bias is unlikely. In addition, this study explored the frequency of consuming sugar as daily versus weekly and did not take into consideration whether the consumption of sugar was during meals or in between. Therefore, future studies may focus on the frequency of sugary diet during different times of the day.

In conclusion, although the relationship between frequency and quantity is difficult to be distinguished, the frequency of consumption of sugary diet could be more important than quantity. However, the interaction relationship between quantity and frequency could better explain the complexity of these two important factors. More attention should be paid to adolescents' diet at school and at home, while dental health professionals should focus their dietary advice on reducing the frequency of sugar intake.

## Figures and Tables

**Table 1:** Description of study participants (*N* = 2262).

*N* (%)	
Gender	
Male	976 (43.1)
Female	1286 (56.9)
Parental education	
Father	
University/college education	950 (42)
No/school education	1312 (58)
Mother	
University/college education	816 (36.1)
No/school education	1446 (63.9)
Decayed teeth	
Yes	1688 (74.6)
No	574 (25.4)
*Mean* *±* *SD*	
Age (years)	14.08 ± 1.3
*DMFT*	
Males	3.60 ± 3.14
Females	4.29 ± 3.44
Total	4 ± 3.3

**Table 2:** Logistic regression models showing the influence of frequency and quantity of consumption of dietary intake on caries prevalence.

Dietary items		Univariate OR (95% CI)	Multivariate OR (95% CI)
Frequency (≤once a week (ref) versus ≥daily)	Fruits	0.9 (0.72–1.11)	
	Biscuits	1.08 (0.89–1.33)	
	Chewing gum	1.25 (1.03–1.52)^*∗*^	1.15 (0.94–1.42)
	Candies	1.25 (1.02–1.53)^*∗*^	1.08 (0.87–1.33)
	Fruit juice	1.26 (1.02–1.55)^*∗*^	1.19 (0.96–1.49
	Sports drinks	1.26 (0.74–2.24)	
	Energy drinks	1.88 (1.22–2.89)^*∗*^	1.37 (0.86–2.18)
	Soft drinks	1.47 (1.21–1.79)^*∗*^	1.33 (1.07–1.65)^*∗*^
	Tea/coffee	1.14 (0.95–1.38)	
	Sweetened milk	1.26 (1.03–1.54)^*∗*^	1.11 (0.90–1.38)
Quantity (≤299 ml/day (ref) versus >299 ml/day)	Fruit juice	0.91 (0.73–1.14)	
	Sports drinks	1 (0.56–1.78)	
	Energy drinks	1.91 (1.24–2.93)^*∗*^	1.55 (0.98–2.45)
	Soft drinks	1.18 (0.98–1.43)	
	Tea/coffee	1.03 (0.79–1.36)	
	Sweetened milk	0.92 (0.68–1.25)	
Frequency-quantity interaction^*∗∗*^	Fruit juice	1.01 (0.99–1.03)	
	Sports drinks	1.02 (0.97–1.06)	
	Energy drinks	1.08 (1.04–1.11)^*∗*^	1.06 (1.02–1.10)^*∗*^
	Soft drinks	1.04 (1.02–1.06)^*∗*^	1.03 (1.01–1.04)^*∗*^
	Tea/coffee	1.00 (0.98–1.02)	
	Sweetened milk	1.03 (1.01–1.05)^*∗*^	1.02 (1.00–1.05)^*∗*^

^*∗*^Statistically significant at *P* < 0.05. ^*∗∗*^Frequency-quantity interaction models were based on six-item graded categorical variables. Factors included in the multivariate model are those showing a significant relationship in univariate models. The multivariate model is adjusted for demographics and oral health practices.

**Table 3:** Linear regression models showing the influence of frequency and quantity of consumption of dietary intake on caries experience (DMFT).

Dietary items		Univariate regression coefficient (95% CI)	Multivariate regression coefficient (95% CI)
Frequency (≤once a week (ref) versus ≥daily)	Fruits	−0.32 (−0.62-(−0.2))^*∗*^	−0.17 (−0.47–0.13)
	Biscuits	0.04 (−0.26–0.33)	
	Chewing gum	0.32 (0.03–0.61)^*∗*^	0.18 (−0.11–0.47)
	Candies	0.31 (0.01–0.61)^*∗*^	0.07 (−0.23–0.38)
	Fruit juice	0.48 (0.17–0.78)^*∗*^	0.48 (0.16–0.79)^*∗*^
	Sports drinks	-0.23 (-1.02–0.56)	
	Energy drinks	0.66 (0.13–1.19)^*∗*^	0.29 (-0.28–0.86)
	Soft drinks	0.50 (0.22–0.78)^*∗*^	0.46 (0.16–0.76)^*∗*^
	Tea/coffee	0.08 (-0.2–0.35)	
	Sweetened milk	0.42 (0.13–0.75)^*∗*^	0.16 (-0.13–0.45)
Quantity (≤299 ml/day (ref) versus >299 ml/day)	Fruit juice	−0.23 (−0.09–0.56)	
	Sports drinks	−0.24 (−1.08–0.60)	
	Energy drinks	0.58 (0.05–1.10)^*∗*^	0.19 (−0.38–0.75)
	Soft drinks	0.18 (−0.09–0.46)	
	Tea/coffee	−0.03 (−0.43–0.36)	
	Sweetened milk	0.29 (-0.17–0.74)	
Frequency-quantity interaction^*∗∗*^	Fruit juice	0.02 (−0.01–0.04)	
	Sports drinks	−0.02 (−0.08–0.03)	
	Energy drinks	0.07 (0.03–0.11)^*∗*^	0.05 (0.01–0.09)^*∗*^
	Soft drinks	0.05 (0.02–0.07)^*∗*^	0.03 (0.01–0.06)^*∗*^
	Tea/coffee	−0.00 (-0.03–0.02)	
	Sweetened milk	0.07 (0.04–0.10)^*∗*^	0.06 (0.03–0.09)^*∗*^

^*∗*^Statistically significant at *P* < 0.05. ^*∗∗*^Frequency-quantity interaction models were based on six-item graded categorical variables. Factors included in the multivariate model are those showing a significant relationship in univariate models. The multivariate model is adjusted for demographics and oral health practices.

## Data Availability

The data used to support the findings of this study are available from the authors upon request.
